# Psychometric properties, validation, and reliability of the Japanese version of the sport anxiety scale-2 for university student athletes

**DOI:** 10.3389/fpsyg.2025.1713901

**Published:** 2025-12-16

**Authors:** Tatsuya Yamaguchi, Shunsuke Takagi, Hikaru Hori, Masaki Nishida, Tomoki Koeda, Takuma Matsumoto, Yujiro Kawata, Tsuneyoshi Ota, Yuji Takazawa

**Affiliations:** 1 Department of Sports Medicine, Faculty of Medicine, Juntendo University, Bunkyo-ku, Tokyo, Japan; 2 Graduate School of Health and Sports Science, Juntendo University, Inzai-shi, Chiba, Japan; 3 Sports Clinic, Department of Sports Medicine, Japan Institute of Sports Sciences, Japan High Performance Sport Center, Kita-ku, Tokyo, Japan; 4 Department of Psychiatry and Behavioral Neurosciences, Graduate School of Institute of Science Tokyo, Institute of Science Tokyo, Bunkyo-ku, Tokyo, Japan; 5 Department of Psychiatry, School of Medicine, Fukuoka University, Johna-ku, Fukuoka, Japan; 6 Faculty of Sport Science, Waseda University, Tokorozawa, Saitama, Japan; 7 Faculty of Health and Sports Science, Juntendo University, Inzai-shi, Chiba, Japan

**Keywords:** competitive anxiety, sport anxiety scale-2 (SAS-2), Japanese athletes, psychometric properties, athlete mental health

## Abstract

**Background:**

Competitive anxiety is a critical factor that affects athletes’ performance and mental well-being. The Sport Anxiety Scale-2 (SAS-2) is a widely used multidimensional measure for sport-specific anxiety, but a validated Japanese version has been unavailable. This study aimed to develop a Japanese version of the SAS-2 and evaluate its reliability and validity among university athletes in Japan.

**Methods:**

After a standard back-translation process, 640 university athletes (mean age 19.1 ± 1.0 years) completed an online survey containing the Japanese SAS-2 and scales for competitive anxiety (Sport Competition Anxiety Test for Adults; SCAT-A), depressive symptoms (Patient Health Questionnaire-9; PHQ-9), and self-esteem (Rosenberg Self-Esteem Scale; RSES). Reliability was assessed through internal consistency and 2-week test–retest reliability (*n* = 140). Construct validity was evaluated using confirmatory factor analysis (CFA), while criterion-related validity was established through correlations.

**Results:**

The Japanese SAS-2 demonstrated excellent internal consistency (Cronbach’s *α* = 0.91) and good test–retest reliability for the total score (ICC = 0.72). Validity was strongly supported by a high correlation with SCAT-A (*r* = 0.74) and expected moderate correlations with depressive symptoms (*r* = 0.42) and self-esteem (*r* = −0.41). The original three-factor structure (somatic anxiety, worry, and concentration disruption) was supported by CFA, with marginal model fit indices (e.g., CFI = 0.884, SRMR = 0.063). Notably, total anxiety scores and all subscale scores were significantly lower among athletes with more years of competitive experience.

**Conclusion:**

The Japanese version of SAS-2 is a reliable and valid instrument for assessing multidimensional competitive anxiety in university athletes. The availability of this tool fills a critical gap in Japanese sports psychology, offering a valuable resource for researchers, clinicians, and coaches. It enables a more nuanced understanding of sport-specific anxiety, helping to identify athletes who need support, facilitate targeted interventions, and ultimately promote athlete mental health and well-being.

## 1 Introduction

Athletes often face unique mental health challenges, including depression, anxiety, and overtraining syndrome (Reardon et al., 2019). Among those, competitive anxiety is a significant psychological factor that affects athletic performance and well-being. Unlike general anxiety, competitive anxiety is defined by situational specificity and temporal characteristics, making its accurate assessment essential for developing targeted psychological interventions (Reardon et al., 2024). Furthermore, competitive anxiety comprises somatic symptoms (e.g., muscle tension, autonomic arousal) and cognitive symptoms (e.g., worry, concentration disruption) (Martens et al., 1990; Vealey, 1990).

The impact of this anxiety on performance is complex, as it is not uniformly negative. Indeed, the relationship between anxiety and athletic performance is best understood as individualized, as described by the Individual Zones of Optimal Functioning (IZOF) model (Ruiz et al., 2015). This model posits that each athlete has a unique “zone” of anxiety intensity where they perform optimally; performance suffers when they are either under-aroused (e.g., complacent, low energy) or over-aroused (e.g., overwhelmed, tense) (Ruiz et al., 2015). For example, an individual athlete like a golfer approaching a critical putt may experience performance-debilitating “choking” when cognitive anxiety is too high, whereas a track sprinter might interpret high somatic arousal as readiness and channel it for an explosive start. In team sports, such as soccer or basketball, anxiety can also be “contagious,” where one player’s visible worry or poor decision-making under pressure can disrupt team cohesion, communication, and tactical execution (Prapavessis and Carron, 1996).

Given this complex and individualized impact, the accurate assessment of competitive anxiety is essential for developing targeted psychological interventions. The Sport Anxiety Scale-2 (SAS-2), developed by Smith et al. (2006), is a multidimensional measure that assesses sport-specific trait anxiety through three subscales: somatic anxiety: So, worry: Wo, and concentration disruption: Co (Smith et al., 2006). The SAS-2 has been validated in multiple countries including Brazil, Korea, Poland, Spain, China, and Indonesia, demonstrating strong psychometric properties in diverse cultural and athletic populations (Dunn and Dunn, 2010; Li et al., 2023; Putra et al., 2021; Ramis et al., 2015; Rawat et al., 2023; Silva-Rocha et al., 2019; Tomczak et al., 2022; Zhang et al., 2023). However, a validated Japanese version of SAS-2 has been lacking, despite increasing awareness of athlete mental health in Japan and the increasing demand for culturally sensitive assessment tools.

The present study aimed to develop a Japanese version of the SAS-2 and evaluate its reliability and validity among university athletes in Japan. Furthermore, we explored associations between SAS-2 scores and related psychological constructs such as self-esteem and depressive symptoms, as well as demographic factors including years of competition and competitive level.

## 2 Methods

### 2.1 Participants

This study was approved by the Ethical Committee Board of the Faculty of Medicine, Juntendo University and conducted following the Helsinki Declaration (Research No. E23-0185). All student-athletes participated voluntarily and provided informed consent online.

The current study was conducted using the online questionnaire platform, where participants accessed the questionnaire by scanning a QR code with their mobile phones. This study was conducted between October 2023 and October 2024 using an anonymous online questionnaire. Participants were recruited from Juntendo University and Waseda University, specifically from the Faculty of Sports and Health Science. Students with regular physical activity, such as participation in sports clubs or athletic teams, were invited to participate during classes or seminars.

We set the inclusion criteria as follows: students aged 18 years or older at the time of consent, who were members of a sports club and had a regular exercise routine, and who gave written consent of their own free will after being fully informed about and understanding the research. Exclusion criteria were as follows: those unable to understand Japanese in the questionnaire, and those with a history of anxiety, depression, or other mental illnesses requiring hospital treatment or hospitalization.

### 2.2 Language adaptation

The adaptation of the language was carried out using the general translation and back-translation process in the field of psychology (Brislin, 1970; Klotz et al., 2023). Briefly, a group of sports psychology, psychology, and psychiatry experts, who were also bilingual Japanese-English translators, translated SAS-2 into Japanese, and another group of Japanese-English bilingual translators, who did not have access to SAS-2, performed back-translation. The implications of each item were maintained by comparing SAS-2 and the back-translation version, and the differences were discussed and agreed upon by the experts. The lead author, TY, who has over 10 years of experience in the fields of sports psychiatry and sports psychology, verified the validity of the translation and back translation. Table 1 shows the Japanese version of SAS-2.

**Table 1:** Japanese-language sport anxiety scale-2 (SAS-2).

No.	Original SAS-2	Japanese SAS-2	Options			
	Guidance in English	Guidance in Japanese	Scale in Japanese			
	Please read each question. Then, circle the number that says how you USUALLY feel before or while you compete in sports. There are no right or wrong answers. Please be as truthful as you can.	それぞれの質問を読んで、あなたが競技の試合前や試合中に、普段どのように感じるか、当てはまる番号に○をつけてください。正解も不正解もありませんので、できるだけ正直に答えてください。	まったくない	少しある	かなりある	非常にある
1.	It is hard to concentrate on the game	試合に集中するのが困難である	1	2	3	4
2.	My body feels tense	身体が緊張する	1	2	3	4
3.	I worry that I will not play well	うまくプレーできないのではないかと心配する	1	2	3	4
4.	It is hard for me to focus on what I am supposed to do	自分のやるべきことに集中するのが困難である	1	2	3	4
5.	I worry that I will let others down	他人を失望させるのではないかと心配する	1	2	3	4
6.	I feel tense in my stomach	競技前、または競技中に胃が緊張する	1	2	3	4
7.	I lose focus on the game	試合に対する集中力が落ちる	1	2	3	4
8.	I worry that I will not play my best	ベストのプレーが出来ないのではないかと心配する	1	2	3	4
9.	I worry that I will play badly	下手なプレーをするのではないかと心配する	1	2	3	4
10.	My muscles feel shaky	筋肉が震える	1	2	3	4
11.	I worry that I will mess up during the game	試合でしくじるのではないかと心配する	1	2	3	4
12.	My stomach feels upset	胃がむかむかする	1	2	3	4
13.	I cannot think clearly during the game	試合の間ずっと明確に考えることができない	1	2	3	4
14.	My muscles feel tight because I am nervous	緊張のため筋肉が硬く感じられる	1	2	3	4
15.	I have a hard time focusing on what my coach tells me to do	コーチにやるように言われることに集中するのが難しい	1	2	3	4

### 2.3 Measures

#### 2.3.1 The sport anxiety scale 2 (SAS-2)

The SAS-2 is a 15-item questionnaire designed to measure the levels of anxiety experienced by athletes before and during competition (Smith et al., 2006). The scale comprises three factors: somatic anxiety, worry, and concentration disruption, each consisting of five items. Participants in this study responded using a four-point Likert scale, where 1 indicated “not at all” and 4 indicated “very much.” Higher scores on the scale indicate higher levels of sports anxiety experienced either before or during competition. We used several related questionnaires to examine the reliability and validity of the Japanese version of SAS-2.

#### 2.3.2 Sports competition anxiety test for adults (SCAT-A)

We used SCAT-A to evaluate the convergent validity of SAS-2 in Japanese. SCAT-A consists of 15 items using a 3-point Likert scale (1 = “Rarely,” 2 = “Sometimes,” and 3 = “Often”). Ten items measure anxiety related symptoms and 5 items are included to reduce response bias and are therefore not scored. A total score is calculated by adding the 10 items, and a higher score indicates a greater likelihood of competitive anxiety. This study applied the Japanese-language SCAT, which has been validated in 10 to 25-year-old Japanese athletes (Endo, 1986). McDonald’s omega for the Japanese-language SCAT in this study was 0.708.

#### 2.3.3 Patient health questionnaire 9 (PHQ-9)

PHQ-9 was developed to assess the presence of depressive symptoms, and consists of nine items scored on a four-point Likert scale (0 = not at all to 3 = nearly every day) (Spitzer et al., 1999; Spitzer et al., 1994). An adapted version with demonstrated validity for the proposed Japanese context was used (cutoff score = 10, sensitivity = 1.00, specificity = 0.98, positive predictive value = 0.97, and negative predictive value = 1.00) (Inagaki et al., 2013; Muramatsu et al., 2007).

#### 2.3.4 Rosenberg self-esteem scale (RSES)

The RSES is a 10-item scale used in many countries, including Japan, to measure self-esteem (Rosenberg, 1965). The RSES was developed by Rosenberg to quantify global positive and negative attitudes toward the self. It consists of 10 items that allow four responses on a Likert scale: strongly agree, agree, disagree, and strongly disagree. These are scored 1, 2, 3 and 4, respectively for negative items, but vice versa for positive items. The total possible scores range from 10 to 40; the higher the score, the higher the level of self-esteem.

We used the Japanese version translated by Mimura and Griffiths (2007). Internal consistency was verified using Cronbach’s alpha coefficient, and the stability of the scale was verified using the retest method, as well as construct validity (factor validity) (Ogihara and Kusumi, 2020; Oshio et al., 2014).

#### 2.3.5 Task and ego orientation in sports questionnaire (TEOSQ)

The Task and Ego Orientation in Sport Questionnaire was used to measure goal orientation (Duda, 1989; Duda and Nicholls, 1992; Li et al., 1996; Lochbaum et al., 2016; Morales-Sanchez et al., 2022). It consists of 13 statements. Seven items concern task orientation, and six items concern ego orientation. The respondent determines to what extent a given statement applies to him/her fiver responses on a Likert scale. (1 = strongly disagree, 5 = strongly agree). The usefulness of the Japanese version has also been confirmed, and this study used the Japanese version developed by Isogai et al. (2003) and Wakayama et al. (2002).

#### 2.3.6 Athletic identity measurement scale (AIMS)

This scale defines athletic identity as the degree of identification with the role of an athlete and scores it on a single factor. In the original study, the scale consisted of 10 items (Brewer and Cornelius, 2001; Brewer et al., 1993), but it is considered appropriate to score it on 7 of the 10 items. It is scored on a 7-point scale from “strongly disagree” to “strongly agree,” with a minimum score of 7 and a maximum of 49, with higher scores indicating a higher level of athletic identity. A Japanese version of the AIMS has already been developed and its reliability and validity have been confirmed (Hagiwara and Isogai, 2014; Hagiwara et al., 2018; Hagiwara et al., 2020).

### 2.4 Statistical analysis

Normality for all continuous variables was assessed via the Shapiro–Wilk test. The Cronbach’s alpha coefficient and McDonald’s omega coefficient were calculated to evaluate the internal consistency of the SAS-2 score, which consists of 15 questions. The bootstrap method (number of iterations: 100,000) was also used to calculate 95% confidence intervals (CI) for each internal consistency index.

Using test–retest data from the SAS-2 score, the intraclass correlation coefficient with a one-way variable effects model [ICC (1, 1)] was calculated as a within-subject reliability measure. Bland–Altman analysis was performed to evaluate the inter-measurement error of the two SAS-2 scores; a Bland–Altman plot was created with the x-axis as the mean of the two SAS-2 scores and the y-axis as the difference between the two measurements. The mean difference and its 95% CI were calculated as fixed error indices. A fixed error was considered present if the 95% CI did not include 0. The correlation coefficient was calculated as a measure of the proportional error.

Correlation analysis with external indices (SCAT-A, TEOSQ, PHQ-9, AIMS, and RSES) was performed to evaluate the criterion-related validity of the SAS-2 score. Pearson’s product–moment correlation and R-square values were calculated. To evaluate the construct validity of the SAS-2 score, a confirmatory factor analysis was performed using three concept factors (somatic anxiety [So], worry [Wo], and concentration-disruption [Co]) and the corresponding questions based on the preliminary hypothesis.

Descriptive statistics were performed on the characteristics of the respondents for the initial survey data. For nominal scales, frequencies and proportions were calculated. For continuous scales, summary statistics (mean, standard deviation [SD], median, interquartile range [IQR], and range) were calculated.

Statistics for each outcome score by years of competition and competition performance will be calculated to evaluate the association. Years of competition were divided into four areas by quartiles. The statistics for each score were calculated for each group of years of competition and athletic performance, and a trend test (linear contrast test in a one-way ANOVA model) was conducted to evaluate the linear association.

All analyzes were performed using R version 4.2.2 (The R Foundation for Statistical Computing). *p* < 0.05 (two-tailed) was considered statistically significant.

## 3 Results

### 3.1 Characteristics of the respondents

A total of 680 students were invited to participate. Of these, 640 cases (278 Individual sports, 279 team sport, 83 non-specific sports) provided valid responses (response rate 94.1%) and were considered the analysis. Forty respondents were excluded due to the absence of regular exercise habits, a history of mental health problems, or refusal to complete the online survey.

Furthermore, a subsample of 140 participants was retested for reliability analysis. All respondents were Japanese university students aged 18–25 years with experience in regular physical activity. The characteristics of the respondents in the analysis population are shown in Table 2. There were 399 males and 241 females, with a mean age of 19.1 years (SD: 1.0), and responses ranged from 18 to 24 years old. The median number of years of competition was 9.0 years, with a quartile range of 6.3 to 12.0 years. The competition levels were 27 (4.2%) recreational levels in hobbies, clubs, and enthusiasts, 150 (23.4%) in district competitions, 150 (23.4%) in regional competitions, 298 cases (46.6%) in national competitions, and 15 (2.3%) in international and world competitions.

**Table 2:** Demographics of all respondents (*n* = 640).

Variables	Attributes and scores	All respondents
Gender (*n*, %)	Male	399, 62.34
	Female	241, 37.66
Age (years)	Mean ± SD	19.1 ± 1.0
	Median [IQR]	19.0 [18.0, 20.0]
	Range	18.0-24.0
Competition history (years)	Mean ± SD	8.7 ± 4.2
	Median [IQR]	9.0 [6.3, 12.0]
	Range	0.0-18.0
Level of competition (*n*, %)	Recreational	27, 4.22
	District	150, 23.44
	Regional	150, 23.44
	National	298, 46.56
	International	15, 2.34
Training hours (hours/week)	Mean ± SD	13.7 ± 7.9
	Median [IQR]	14.0 [9.0, 20.0]
	Range	0.0-70.0
SAS-2 Total score	Mean ± SD	26.5 ± 7.5
	Median [IQR]	26.0 [21.0, 31.0]
	Range	15.0-53.0
SAS-2 score: So	Mean ± SD	8.5 ± 2.7
	Median [IQR]	8.0 [6.0, 10.0]
	Range	5.0-20.0
SAS-2 score: Wo	Mean ± SD	10.5 ± 3.7
	Median [IQR]	10.0 [8.0, 12.0]
	Range	5.0-20.0
SAS-2 score: Co	Mean ± SD	7.6 ± 2.4
	Median [IQR]	7.0 [5.0, 9.0]
	Range	5.0-18.0
SCAT-A	Mean ± SD	19.7 ± 4.3
	Median [IQR]	20.0 [17.0, 23.0]
	Range	10.0-30.0
PHQ-9	Mean ± SD	4.7 ± 4.3
	Median [IQR]	4.0 [1.0, 7.0]
	Range	0.0-27.0
RSES	Mean ± SD	26.2 ± 4.6
	Median [IQR]	26.0 [24.0, 29.0]
	Range	11.0-40.0
TEOSQ: Total score	Mean ± SD	54.6 ± 9.5
	Median [IQR]	56.0 [50.0, 62.0]
	Range	13.0-65.0
TEOSQ: Ego	Mean ± SD	4.1 ± 0.8
	Median [IQR]	4.2 [3.7, 4.8]
	Range	1.0-5.0
TEOSQ: Task	Mean ± SD	4.3 ± 0.7
	Median [IQR]	4.4 [4.0, 4.9]
	Range	1.0-5.0
AIMS	Mean ± SD	36.9 ± 7.8
	Median [IQR]	38.0 [32.0, 43.0]
	Range	7.0-49.0

SD: standard deviation, IQR: Inter-quartile range [25, 75%]. Range: Minimum and maximum values. So: Somatic anxiety, Wo: Worry, Co: Concentration Disruption. SCAT-A: Sports Competition Anxiety Test for Adults. PHQ-9: Patient Health Questionnaire-9. RSES: Rosenberg Self-Esteem Scale. TEOSQ: Task and Ego Orientation in Sports Questionnaire. AIMS: Athletic Identity Measurement Scale.

### 3.2 Reliability and validity assessment of the SAS-2 score

**Figure 1:**
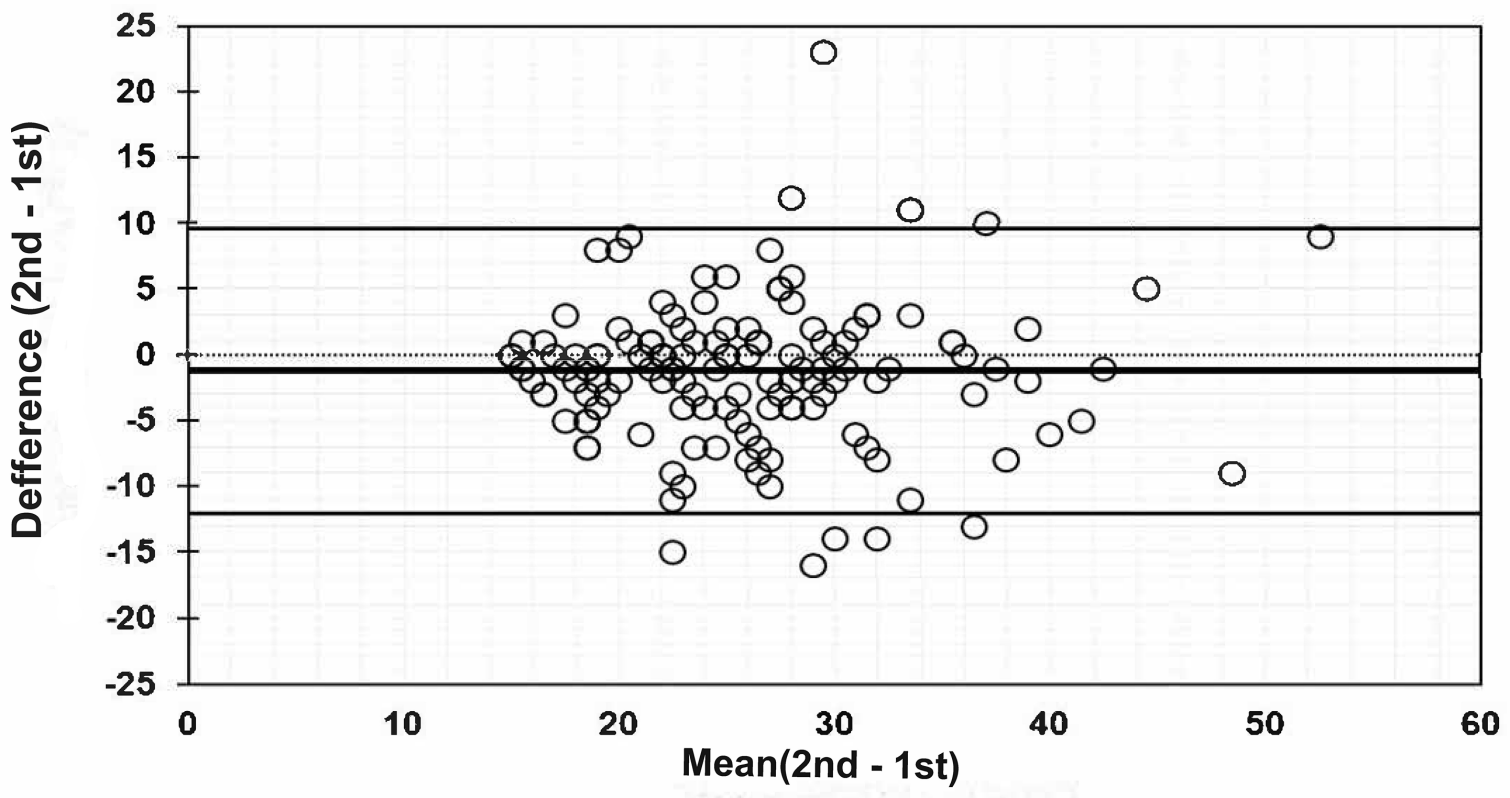
Bland–Altman plot: 2nd total score 1st total score.

The internal consistency index of the SAS-2 score was 0.911 [95%CI: 0.900, 0.921] for Cronbach’s alpha and 0.914 [0.902, 0.924] for McDonald’s omega, with both indexes exceeding 0.9 (Table 3). The ICC (1, 1) as a within-subject reliability measure was 0.722 [0.633, 0.793] for the SAS-2 total score. The sub-scores of SAS-2 were 0.666 [0.563, 0.749] for the So index, 0.650 [0.543, 0.736] for the Wo index, and 0.655 [0.549, 0.740] for the Co index (Table 4). A Bland–Altman plot of the error between two measurements of the SAS-2 score is shown in Table 5 and Figure 1. The difference in mean was −1.243 [−2.164, −0.322] points (second measurement − first measurement), detected as a statistically significant fixed error. No statistically significant proportional errors were detected (correlation coefficient = 0.015, *p* = 0.864).

**Table 3:** The internal consistency index of the SAS-2 score.

Coefficient	Value	95%CI
Cronbach’s alpha	0.911	0.900, 0.921
McDonald’s omega	0.914	0.902, 0.924

SAS-2: Number of questions = 15. Number of respondents: *n* = 640. 95%CI: 95% confidence interval.

**Table 4:** SAS-2 test–retest reliability: reliability assessment.

SAS-2 scores	ICC (1, 1)	
	Value	95%CI
Total score	0.722	0.633, 0.793
Somatic anxiety	0.666	0.563, 0.749
Worry	0.650	0.543, 0.736
Concentration disruption	0.655	0.549, 0.740

Number of respondents: *n* = 140. ICC(1, 1): intraclass correlation coefficient [univariate effect model].

**Table 5:** Bland Altman analysis for SAS-2 scores.

Scores	Fixed error			Proportional error		
	Difference of mean	95% CI	Existence of error	Correlation coefficient	*p*-value	Existence of error
2nd – 1st	−1.243	−2.164, −0.322	Yes	0.015	0.864	No

CI, confidence interval. Number of respondents: *n* = 140.

The results of the correlation analysis with external indicators are shown in Table 6. For the SAS-2 Total score, a high correlation of 0.741 (*R*^2^ = 0.549) was found with SCAT-A. It also showed a moderate correlation with PHQ-9 score (*r* = 0.418, *R*^2^ = 0.175) and RSES (*r* = −0.412, *R*^2^ = 0.170). On the other hand, no correlation was observed with TEOSQ and AIMS (correlation coefficient less than 0.2). Each sub-score of the SAS-2 was also correlated with the SCAT-A, PHQ-9, and RSES, as was the total score, although the strength of the correlation varied.

**Table 6:** Results of correlation analysis of external indicators in SAS-2.

Psychological questionnaire		SAS-2 total score			SAS-2 score: So			SAS-2 score: Wo			SAS-2 score: Co		
		*r*	*P*-value	*R* ^2^	*r*	*P*-value	*R* ^2^	*r*	*P*-value	*R* ^2^	*r*	*P*-value	*R* ^2^
SCAT-A		**0.741**	**<0.001**	0.549	**0.674**	**<0.001**	0.454	**0.701**	**<0.001**	0.491	0.474	**<0.001**	0.225
PHQ-9		0.418	**<0.001**	0.175	0.302	**<0.001**	0.091	0.370	**<0.001**	0.137	0.397	**<0.001**	0.158
RSES		−0.412	**<0.001**	0.170	−0.284	**<0.001**	0.080	−0.418	**<0.001**	0.175	−0.325	**<0.001**	0.105
TEOSQ	Total	0.036	0.366	0.001	0.014	0.727	0.000	0.080	0.043	0.006	−0.027	0.489	0.001
	Ego	0.044	0.270	0.002	0.005	0.890	0.000	0.100	0.012	0.010	−0.023	0.558	0.001
	Task	0.024	0.545	0.001	0.020	0.612	0.000	0.052	0.190	0.003	−0.028	0.480	0.001
AIMS		−0.049	**0.213**	0.002	−0.013	0.744	0.000	−0.023	0.567	0.001	−0.105	**0.008**	0.011

So: Somatic anxiety, Wo: Worry, Co: Concentration Disruption. SCAT-A: Sports Competition Anxiety Test for Adults. PHQ-9: Patient Health Questionnaire-9. RSES: Rosenberg Self-Esteem Scale. TEOSQ: Task and Ego Orientation in Sports Questionnaire. AIMS: Athletic Identity Measurement Scale. Bold text indicates a *p*-value that is clearly significantly lower.

**Figure 2:**
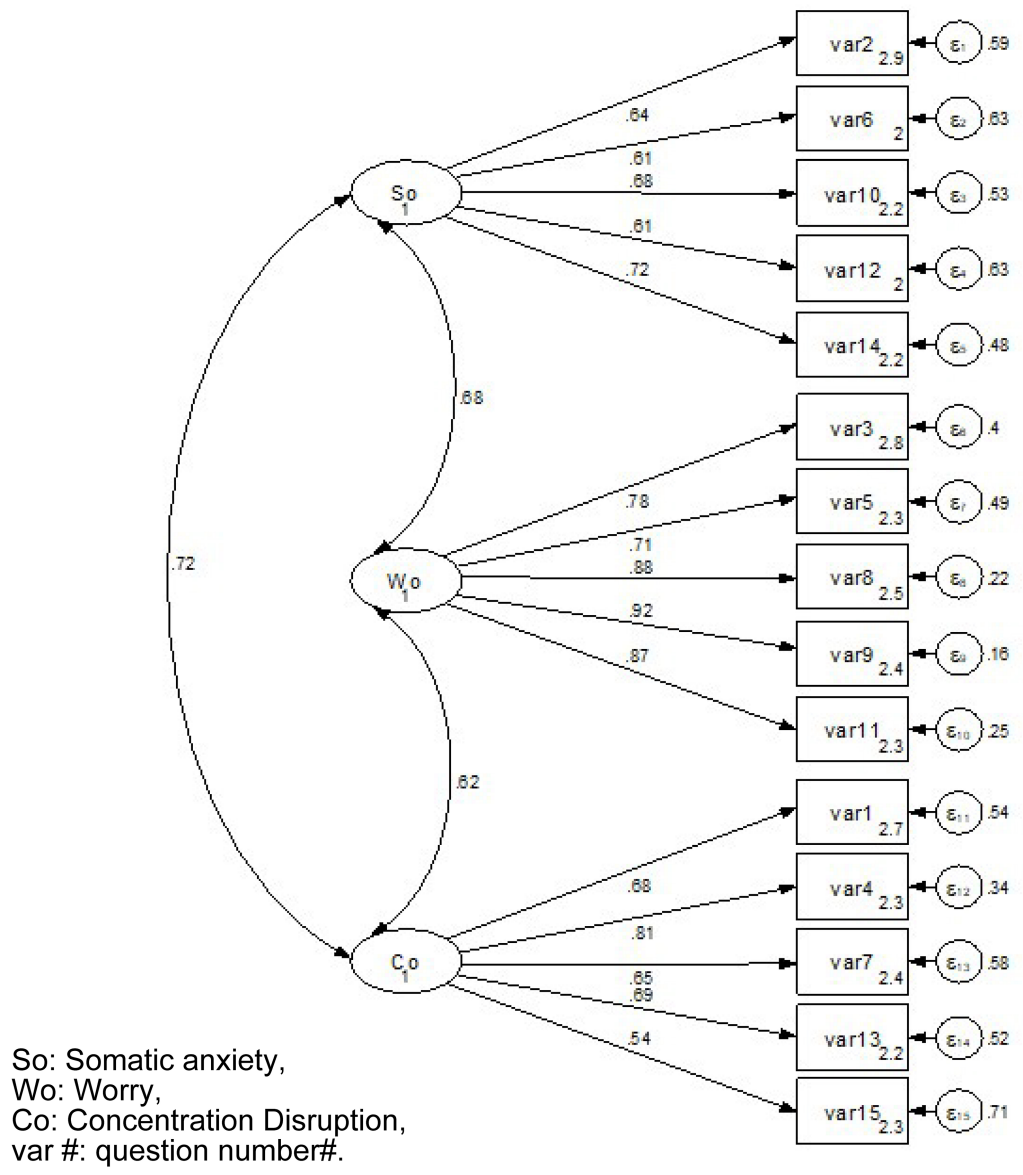
The result of confirmatory factor analysis.

The results of the confirmatory factor analysis are shown in Figure 2 and Table 7. Correlations were confirmed between each latent factor (So, Wo, and Co) and each of the questions tied to it. In the goodness-of-fit index, the CFI was 0.884 and the AGFI was 0.853. The RMSEA and SRMR were 0.105 and 0.063, respectively.

**Table 7:** Results of analysis on structural concept validity.

Analysis	Value
Chi-square (df)	696.112 (87)
R-squared	0.994
CFI	0.884
TLI	0.860
RMSEA [90%CI]	0.105 [0.097, 0.112]
SRMR	0.063
GFI	0.870
AGFI	0.853

CFI: Comparative fit index; TLI: Tucker-Lewis index; RMSEA: Root mean squared error of approximation; SRMR: Standardized root mean squared residual; GFI: Goodness-of-fit Index; AGFI: Adjusted goodness-of-fit index.

### 3.3 Correlation between evaluation items and years of competition and performance

The relationship between years of competition and endpoints is shown in Table 8. The SAS-2 score showed a statistically significant trend toward decreasing with years of competition for both total score and all sub-scores. The SAS-2 total score was 27.9 (SD: 7.3) for the shortest group (6 years or less), while it was 24.7 (7.1) for the longest group (12 years or more). SCAT-A scores also showed a significant trend toward lower scores with longer years of competition [the shortest group: 20.5 (4.2), the longest group: 18.5 (4.3), *p* < 0.001 for trend]. Furthermore, for AIMS, there was a significant trend for scores to increase with years of competition [the shortest group: 34.9 (8.7), the longest group: 37.5 (7.4), *p* = 0.004 for trend]. No statistically significant linear correlations were found for the other endpoints.

**Table 8:** Results of the relationship between years of competition and each score.

Psychological questionnaire	Variables	Q1 [≤ 6y]	Q2 [7, 8y]	Q3 [9, 10, 11y]	Q4 [12y ≤]	*P*-value for trend
		*n* = 160	*n* = 120	*n* = 178	*n* = 182	
SAS-2 Total score	Mean ± SD	27.9 ± 7.3	26.9 ± 7.5	26.9 ± 7.8	24.7 ± 7.1	**<0.001**
	Median [IQR]	28.0 [22.0, 31.8]	26.0 [21.0, 32.0]	25.0 [21.0, 31.0]	24.0 [19.0, 29.3]	
	Range	15.0–53.0	15.0–47.0	15.0–53.0	15.0–51.0	
SAS-2 score: So	Mean ± SD	8.7 ± 2.7	8.8 ± 2.7	8.5 ± 2.9	8.1 ± 2.6	**0.035**
	Median [IQR]	8.0 [7.0, 10.0]	8.0 [7.0, 10.0]	8.0 [6.0, 10.0]	7.5 [6.0, 9.3]	
	range	5.0–20.0	5.0–18.0	5.0–19.0	5.0–18.0	
SAS-2 score: Wo	Mean ± SD	11.2 ± 3.8	10.5 ± 3.6	10.8 ± 3.7	9.5 ± 3.5	**<0.001**
	Median [IQR]	10.0 [9.0, 14.0]	10.0 [8.0, 12.0]	10.0 [8.8, 13.0]	9.5 [6.8, 11.0]	
	Range	5.0–20.0	5.0–20.0	5.0–20.0	5.0–20.0	
SAS-2 score: Co	Mean ± SD	8.0 ± 2.2	7.7 ± 2.5	7.6 ± 2.6	7.0 ± 2.2	**<0.001**
	Median [IQR]	8.0 [6.0, 10.0]	7.0 [5.0, 9.8]	7.0 [5.0, 10.0]	7.0 [5.0, 8.0]	
	Range	5.0–14.0	5.0–17.0	5.0–16.0	5.0–18.0	
SCAT	Mean ± SD	20.5 ± 4.2	20.1 ± 4.2	20.0 ± 4.4	18.5 ± 4.3	**<0.001**
	Median [IQR]	21.0 [18.0, 24.0]	20.0 [17.0, 23.0]	20.0 [17.0, 24.0]	19.0 [15.0, 22.0]	
	Range	11.0–30.0	10.0–28.0	10.0–30.0	10.0–28.0	
PHQ-9	Mean ± SD	4.7 ± 3.9	4.9 ± 4.5	5.3 ± 4.9	3.9 ± 3.9	0.154
	Median [IQR]	4.0 [2.0, 7.8]	4.0 [2.0, 7.0]	4.0 [2.0, 8.0]	3.0 [1.0, 6.0]	
	Range	0.0–16.0	0.0–21.0	0.0–24.0	0.0–27.0	
RSES	Mean ± SD	25.9 ± 4.6	26.0 ± 4.6	25.9 ± 5.1	26.9 ± 4.1	0.069
	Median [IQR]	25.0 [23.0, 28.0]	26.0 [23.0, 29.0]	26.0 [23.0, 29.0]	27.0 [24.0, 29.0]	
	Range	13.0–39.0	13.0–40.0	11.0–39.0	18.0–40.0	
TEOSQ: Total score	Mean ± SD	54.6 ± 10.0	53.2 ± 10.8	56.6 ± 8.1	53.5 ± 9.1	0.902
	Median [IQR]	56.0 [51.0, 62.0]	55.0 [48.0, 62.0]	59.0 [52.0, 63.3]	54.0 [49.0, 61.0]	
	Range	13.0–65.0	13.0–65.0	13.0–65.0	13.0–65.0	
TEOSQ: Ego	Mean ± SD	4.1 ± 0.8	4.0 ± 0.9	4.3 ± 0.7	4.0 ± 0.8	0.591
	Median [IQR]	4.2 [3.8, 4.8]	4.2 [3.4, 4.8]	4.5 [3.8, 5.0]	4.0 [3.7, 4.7]	
	Range	1.0–5.0	1.0–5.0	1.0–5.0	1.0–5.0	
TEOSQ: Task	Mean ± SD	4.2 ± 0.8	4.2 ± 0.8	4.4 ± 0.6	4.2 ± 0.7	0.776
	Median [IQR]	4.4 [4.0, 4.9]	4.3 [3.9, 4.9]	4.6 [4.1, 4.9]	4.3 [3.9, 4.9]	
	Range	1.0–5.0	1.0–5.0	1.0–5.0	1.0–5.0	
AIMS	Mean ± SD	34.9 ± 8.7	37.5 ± 7.7	37.6 ± 7.4	37.5 ± 7.4	**0.004**
	Median [IQR]	37.0 [29.0, 41.8]	39.0 [32.3, 43.8]	39.0 [33.0, 43.0]	39.0 [33.0, 43.0]	
	Range	7.0–49.0	17.0–49.0	12.0–49.0	14.0–49.0	

SD: standard deviation, IQR: Inter-quartile range [25, 75%]. range: Minimum and maximum values. Years of competition were divided into four zones by quartiles (Q1 ~ Q4). So: Somatic anxiety, Wo: Worry, Co: Concentration Disruption. SCAT-A: Sports Competition Anxiety Test for Adults. PHQ-9: Patient Health Questionnaire-9. RSES: Rosenberg Self-Esteem Scale. TEOSQ: Task and Ego Orientation in Sports Questionnaire. AIMS: Athletic Identity Measurement Scale. Bold text indicates a *p*-value that is clearly significantly lower.

The relationship between competitive level and endpoints is shown in Table 9. The SAS-2 sub-scores for worry (Wo) showed a statistically significant decreasing trend with competitive level [Recreational group: 10.3 (3.1), world and international competitions group: 9.1 (4.1), *p* = 0.044 for trend]. Furthermore, for AIMS, there was a significant trend for scores to increase with competitive level [Recreational group: 29.5 (8.3), world and international competitions group: 39.6 (7.6), *p* < 0.044 for trend]. No statistically significant linear correlations were found for the other endpoints.

**Table 9:** Results of the relationship between competition level and each score.

Levels of competition		Recreational	District	Regional	National	International	P-value for trend
		*n* = 27	*n* = 150	*n* = 150	*n* = 298	*n* = 15	
SAS-2 Total score	Mean ± SD	26.7 ± 7.3	27.1 ± 8.0	27.2 ± 8.0	26.1 ± 7.0	22.8 ± 6.9	0.060
	Median [IQR]	28.0 [21.0, 33.0]	26.0 [21.0, 32.0]	26.0 [21.0, 31.3]	25.0 [21.0, 30.0]	23.0 [17.0, 27.0]	
	Range	15.0–41.0	15.0–53.0	15.0–53.0	15.0–51.0	15.0–37.0	
SAS-2 score: So	Mean ± SD	8.9 ± 2.7	8.5 ± 2.9	8.6 ± 3.0	8.5 ± 2.5	7.1 ± 1.8	0.380
	Median [IQR]	8.0 [6.0, 10.0]	8.0 [6.0, 10.0]	8.0 [6.0, 10.0]	8.0 [7.0, 10.0]	6.0 [6.0, 9.0]	
	Range	5.0–14.0	5.0–19.0	5.0–20.0	5.0–18.0	5.0–10.0	
SAS-2 score: Wo	Mean ± SD	10.3 ± 3.1	10.8 ± 3.9	11.1 ± 4.0	10.1 ± 3.4	9.1 ± 4.1	**0.044**
	Median [IQR]	10.0 [8.0, 13.0]	10.0 [8.0, 13.0]	10.0 [8.8, 13.3]	10.0 [8.0, 12.0]	9.0 [5.0, 11.0]	
	Range	5.0–15.0	5.0–20.0	5.0–20.0	5.0–20.0	5.0–18.0	
SAS-2 score: Co	Mean ± SD	7.5 ± 2.4	7.9 ± 2.5	7.6 ± 2.5	7.4 ± 2.3	6.7 ± 2.2	0.074
	Median [IQR]	8.0 [5.0, 9.0]	8.0 [6.0, 10.0]	7.0 [5.0, 9.0]	7.0 [5.0, 9.0]	5.0 [5.0, 9.0]	
	Range	5.0–13.0	5.0–16.0	5.0–17.0	5.0–18.0	5.0–10.0	
SCAT-A	Mean ± SD	19.4 ± 4.6	19.7 ± 4.5	20.3 ± 4.4	19.6 ± 4.2	18.0 ± 3.9	0.402
	Median [IQR]	20.0 [15.0, 22.0]	19.5 [17.0, 23.0]	21.0 [17.8, 24.0]	20.0 [17.0, 22.0]	19.0 [15.0, 22.0]	
	Range	11.0–27.0	10.0–29.0	10.0–30.0	10.0–30.0	11.0–23.0	
PHQ-9	Mean ± SD	5.8 ± 4.3	4.8 ± 4.7	4.2 ± 4.0	4.8 ± 4.3	4.0 ± 3.4	0.581
	Median [IQR]	5.0 [2.0, 9.0]	4.0 [1.0, 7.0]	3.0 [2.0, 6.0]	4.0 [2.0, 7.0]	4.0 [1.0, 6.0]	
	Range	0.0–13.0	0.0–21.0	0.0–21.0	0.0–27.0	0.0–11.0	
RSES	Mean ± SD	24.0 ± 4.8	26.2 ± 4.7	26.5 ± 4.9	26.3 ± 4.4	24.9 ± 4.6	0.259
	Median [IQR]	24.0 [21.0, 28.0]	26.0 [24.0, 30.0]	27.0 [23.8, 29.0]	26.0 [24.0, 29.0]	23.0 [21.0, 29.0]	
	Range	15.0–35.0	13.0–40.0	13.0–40.0	11.0–40.0	20.0–35.0	
TEOSQ: Total score	Mean ± SD	51.1 ± 10.5	54.3 ± 10.9	55.4 ± 8.8	54.6 ± 8.7	53.7 ± 13.1	0.322
	Median [IQR]	54.0 [46.0, 58.0]	56.5 [51.0, 62.0]	56.0 [50.8, 63.0]	55.5 [50.0, 62.0]	56.0 [50.0, 65.0]	
	Range	13.0–65.0	13.0–65.0	13.0–65.0	13.0–65.0	17.0–65.0	
TEOSQ: Ego	Mean ± SD	3.8 ± 0.9	4.1 ± 0.9	4.2 ± 0.8	4.1 ± 0.8	4.0 ± 1.0	0.393
	Median [IQR]	4.0 [3.2, 4.5]	4.2 [3.8, 4.8]	4.3 [3.8, 5.0]	4.2 [3.7, 4.8]	4.2 [3.2, 5.0]	
	Range	1.0–5.0	1.0–5.0	1.0–5.0	1.0–5.0	1.5–5.0	
TEOSQ: Task	Mean ± SD	4.0 ± 0.8	4.2 ± 0.8	4.3 ± 0.7	4.3 ± 0.7	4.3 ± 1.1	0.319
	Median [IQR]	4.0 [3.9, 4.7]	4.4 [4.0, 4.9]	4.4 [4.0, 4.9]	4.3 [4.0, 4.9]	4.7 [4.0, 5.0]	
	Range	1.0–5.0	1.0–5.0	1.0–5.0	1.0–5.0	1.1–5.0	
AIMS	Mean ± SD	29.5 ± 8.3	36.1 ± 8.0	35.3 ± 8.3	38.5 ± 6.9	39.6 ± 7.6	**<0.001**
	Median [IQR]	29.0 [23.0, 36.0]	37.5 [32.0, 42.0]	36.0 [29.8, 41.3]	40.0 [34.0, 43.0]	43.0 [31.0, 46.0]	
	Range	11.0–42.0	12.0–49.0	7.0–49.0	17.0–49.0	28.0–49.0	

SD: standard deviation, IQR: Inter-quartile range [25, 75%]. range: Minimum and maximum values. So: Somatic anxiety, Wo: Worry, Co: Concentration Disruption. SCAT-A: Sports Competition Anxiety Test for Adults. PHQ-9: Patient Health Questionnaire-9. RSES: Rosenberg Self-Esteem Scale. TEOSQ: Task and Ego Orientation in Sports Questionnaire. AIMS: Athletic Identity Measurement Scale. Bold text indicates a *p*-value that is clearly significantly lower.

## 4 Discussion

This study aimed to develop a Japanese version of the SAS-2 and to evaluate its psychometric properties among university athletes in Japan. The primary finding of this research is that the Japanese version of SAS-2 is a reliable and valid instrument for assessing multidimensional competitive anxiety in this population. The scale demonstrated excellent internal consistency (Cronbach’s alpha = 0.911; McDonald’s omega = 0.914), good test–retest reliability (ICC = 0.722) comparable to other language versions of SAS-2 (Ramis et al., 2015), and strong evidence of both convergent and criterion-related validity. These results are consistent with findings from previous international validation studies (Akram et al., 2024; Li et al., 2023; Tomczak et al., 2022).

The psychometric properties of Japanese SAS-2 are largely consistent with previous validation studies conducted in diverse cultural contexts, including Brazil, Spain, and China (Li et al., 2021; Ramis et al., 2015; Silva-Rocha and Osorio, 2017). Criterion-related validity was substantiated through significant correlations (0.741) with SCAT-A, demonstrating that SAS-2 effectively measures competitive anxiety similarly to established instruments (Silva-Rocha et al., 2019; Smith et al., 2006).

Furthermore, the study revealed the relationship between competitive anxiety and other key psychological variables. The moderate positive correlation between SAS-2 scores and depressive symptoms (measured by PHQ-9) and the moderate negative correlation with self-esteem (measured by RSES) align with established literature linking anxiety to broader mental health challenges and self-perception in athletes (Beisecker et al., 2024; Vaughan et al., 2020; Weber et al., 2023). These findings underscore the importance of assessing competitive anxiety not in isolation, but as a potential indicator of larger psychological distress. Interestingly, no significant correlations were found with goal orientation (TEOSQ) or athletic identity (AIMS). This suggests that an athlete’s anxiety experience may be district from their motivational orientation (task vs. ego) and the degree to which they identify with their athletic role (Jamshidi et al., 2011).

Confirmatory factor analysis supported the three-factor structure proposed by Smith et al. (2006), although fit indices such as CFI (0.884) and RMSEA (0.105) were marginally lower than recommended thresholds. These results are similar to previous adaptations (Hashim et al., 2017; Li et al., 2023; Silva-Rocha and Osorio, 2017; Tomczak et al., 2022). This may suggest minor cultural nuances in how Japanese athletes interpret or experience the components of anxiety, which could affect the original three-factor structure. Future research could use exploratory factor analysis or qualitative methods to delve deeper into the unique structure of sport anxiety in the Japanese context.

The study further identified the significant negative relationship between competitive experience and anxiety levels. Athletes with more years of competition reported significantly lower total SAS-2 scores, a trend also observed with SCAT-A. This may suggest that experience equips athletes with more effective coping mechanisms or leads to habituation to competitive pressures. Similarly, athletes competing at higher levels reported significantly lower levels of the ‘Worry’ subscale. This finding may suggest that elite athletes employ more effective cognitive strategies to manage worry, or that an inherent capacity to regulate worry serves as a selective factor for achieving higher levels of competition. On the contrary, athletic identity (AIMS score) increased with both years of competition and level of performance, painting a developmental picture where athletes become more secure in their role over time, which may in turn help decrease anxiety (Claes et al., 2025; Geary et al., 2025; Hagiwara et al., 2020; Zhang et al., 2024).

Despite these strengths, some limitations should be acknowledged. The cross-sectional design limits causal inference, necessitating longitudinal studies for more robust conclusions. Firstly, the sample was drawn exclusively from university students (Contreras et al., 2023; Sanfilippo et al., 2024) at two institutions, which can limit the generalizability of the findings. This university population represents a unique developmental stage, bridging amateur and high-performance sport, but their anxiety profiles may differ significantly from other populations. For instance, professional athletes face anxieties directly linked to livelihood, contracts, and intense public scrutiny, which may manifest differently than the academic and athletic pressures faced by university athletes (Li et al., 2021; Rice et al., 2016). Conversely, younger adolescent or amateur adult athletes may experience anxiety related to skill acquisition, developmental challenges, or parental expectations (Pluhar et al., 2019). The applicability of the SAS-2’s structure and the established norms may not directly transfer to these distinct groups. Future studies should aim to validate Japanese SAS-2 across a more diverse range of ages, competition levels (e.g., professional, high-school, recreational), and sport types (Mesquita Vieira et al., 2025; Silva-Rocha et al., 2019; Vaughan et al., 2020). Secondly, the reliance on self-report measures introduces the possibility of response bias. Finally, the test–retest analysis revealed a small but statistically significant fixed error, suggesting a potential practice or familiarization effect. While this did not compromise the overall good reliability, it is a factor to consider in longitudinal applications of the scale. Future research should utilize Japanese SAS-2 to track anxiety longitudinally across a competitive season, to evaluate the effectiveness of psychological interventions, and to further explore its relationship with performance outcomes.

Furthermore, a crucial consideration for the practical application of the Japanese SAS-2 is its utility across both individual and team sports, both of which were included in this validation. The SAS-2 assesses an athlete’s individual anxiety traits, making it broadly applicable. However, the interpretation and source of that anxiety may differ significantly between sport types. For an individual sport athlete (e.g., track and field, golf), high ‘Worry’ scores might relate primarily to personal failure or outcome pressures. In contrast, for a team sport athlete (e.g., basketball, soccer), ‘Worry’ may be strongly linked to social-evaluative concerns, such as fear of letting teammates down, being negatively judged by peers, or disrupting team cohesion (Prapavessis and Carron, 1996). Coaches and sports psychologists should consider these contextual factors when interpreting profiles. Future research should investigate whether the SAS-2 subscales function differently or relate to performance in unique ways for team versus individual sport athletes within the Japanese context.

## 5 Conclusion

In conclusion, this study provides strong evidence that the newly developed Japanese version of the SAS-2 is a reliable and valid multidimensional instrument for measuring competitive anxiety in university athletes. It successfully fills a critical gap in sport psychology assessment tools available in Japan. The availability of this culturally adapted scale is a significant step forward, providing researchers, sports psychologists, and coaches with a valuable tool to better understand, identify, and support athletes experiencing competitive anxiety, ultimately contributing to the promotion of mental health and well-being in the Japanese athletic community. Future research should explore longitudinal changes and extend validation to broader athletic populations.

## Data Availability

The original contributions presented in the study are included in the article/supplementary material, further inquiries can be directed to the corresponding author.
